# Diseases of the Intestines

**Published:** 1906-03-03

**Authors:** 


### Progress in Medicine and Surgery.

### DISEASES OF THE INTESTINES.

**Membranous Colitis.**—This condition has been particularly studied on the Continent, and at Plombières and other health resorts special hydrotherapeutic treatment has been elaborated, and a large amount of statistical data accumulated. Hale White,[1] in a review of sixty cases, states that his series is the first published in this country. His statistical results are generally in harmony with those of the Continental writers. Thus he finds that the condition is far commoner in women than in men, that it is usually found among the wealthier classes, and that it is rarely found under the age of twenty or over that of forty, the commonest period for its appearance being between twenty and thirty. Unless dependent on or complicated by some other disease, such as cancer, membranous colitis seldom causes death, and that, at any rate, a large proportion recover. In Hale White's series 50 per cent. are considered cured, but this is no doubt a low percentage, as the patients were all seen in consultation, and consequently contained a high proportion of severe and protracted cases. Hale White considers that the disease is usually more severe in women than in men, and that in any given case the prognosis will largely depend upon the length of time the disease has gone on. The symptoms are not dealt with at any great length, as they have often been described; some interesting analyses of the intestinal sand (consisting chiefly of calcium phosphate) sometimes passed by these patients is given. From Hale White's account, and from those of other writers, there certainly seems some difficulty in drawing a clear dividing line between cases of temporary constipation in which some membrane is evacuated, and mild cases of membranous colitis. Some chronic cases associated with agonising pain are easily diagnosed as membranous colitis, but whether the condition is altogether *sui generis*, or merely an exaggerated form of constipation, seems at least an open question. The association of membranous colitis with enteroptosis is not one on which Hale White lays as much stress as do certain foreign writers, but he thinks coincident disease of the female pelvic organs of considerable frequency and importance. The association with appendicitis, as Hale White is careful to point out, is only of importance if it can be shown that the appendicitis precedes and causes the membranous colitis; in these operation for appendicitis may be valuable. Where the involvement of the appendix is secondary, its removal can do no good. From a pathological point of view Hale White, somewhat in opposition to the Continental writers, is inclined to regard the condition as a local one, and to ascribe the well-known nervous symptoms to the primary disturbance in the digestive tract. As to treatment, the main indication is to keep the large intestine empty. In Hale White's experience many cases are cured by castor oil, taken early every morning—if necessary for years. Sulphate of magnesium or calomel over night may be substituted. Intractable cases must be given large enemata of water at 100° F., or, better still, should be sent to Plombières. High frequency currents have occasionally done good, but have often been useless. As a last resort right-sided colotomy should be performed, but there are well-known disadvantages attached to this operation. Hale White cautions against starving the patient; he believes an ordinary, plain, simple diet to be the best. Intestinal antiseptics and astringents are useless. The patient must be protected from cold, and the treatment persevered in for months, as improvement is often very tardy.

[1] Lancet, Oct. 28, 1905, p. 1229.

(*To be continued.*)

---

